# Changes in sex hormones and their interactions are related to pain perception between different menstrual subphases

**DOI:** 10.1152/ajpregu.00275.2022

**Published:** 2023-07-17

**Authors:** Luyao Zhang, Ying Zhao, Xinmin Liu, Juan Chen, Mingyang Sun, Jiaqiang Zhang, Wei Zhang

**Affiliations:** ^1^Department of Anesthesiology and Perioperative Medicine, Henan Provincial People’s Hospital, People’s Hospital of Zhengzhou University, Zhengzhou, Henan, People’s Republic of China; ^2^Department of Ultrasound, Henan Provincial People’s Hospital, People’s Hospital of Zhengzhou University, Zhengzhou, Henan, People’s Republic of China

**Keywords:** absolute change, menstrual cycle, pain perception, relative change, sex hormones

## Abstract

Whether sex hormones are related to pain perception across the menstrual cycle is unclear. We examined changes in experimental pain perception in healthy young females between the early to midfollicular subphase (emF) and the midluteal subphase (mL) and explored the role of sex hormones. Sixty-six participants were involved in the study. We tested pressure pain, cold pain, ischemic pain, and needle pain, while at the same time we measured sex hormones levels in two menstrual subphases. Only the right ulna pressure test showed a significant reduction in pain threshold (PPTh3) during the mL. The absolute change of PPTh3 (PPTh3_mL_ − PPTh3_emF_) was related to the absolute change of prolactin. The relative change of the range of pain tolerance for pressure pain of the right ulna (RPT3_rc_) was related to the relative change of progesterone (P_rc_) and estradiol (E2_rc_) levels, and the interaction effects showed that at P_rc_ ≤ 30, E2_rc_ was positively correlated with RPT3_rc_. The same, the relative change of pressure pain tolerance of the pulp of the middle finger on the right hand (PPTo4_rc_) was related to E2_rc_ and P_rc_, and the results of the interaction between E2_rc_ and P_rc_ suggest that when E2_rc_ is ≤0.8, P_rc_ is positively correlated with PPTo4_rc_. Two different formulas were applied in this study and showed inconsistent results. Most pain tests showed no difference between the two subphases of the menstrual cycle. Only the relative changes of the PPTo4 and RPT3 are related to the E2_rc_ and P_rc_, respectively, between menstrual subphases in an interactive way in healthy young women.

## INTRODUCTION

In pain research, sex differences have been studied extensively. Many studies have shown that women are more likely to experience many specific clinical pain conditions than men and are more sensitive to acute experimental pain (1, 2). Therefore, it is particularly important to look for the mechanisms that underlie pain in women.

Female sex hormones may alter endogenous pain modulation and analgesia (3–8) and have been demonstrated to interact with nociceptive processes at multiple levels of the peripheral and central nervous system (9–13). Up to now, there is a lack of consistency regarding the relationship between these hormones and pain perception, and the underlying mechanisms and the precise role of sex hormones in modulating nociception and pain are complex and not fully understood (1, 14, 15). Estrogen and progesterone have been observed to generate both antinociceptive and pronociceptive effects on pain pathway (1, 13, 16), suggesting that the effect of these hormones on pain is complex. Previous experiments on animals have shown that progesterone and estradiol interact in the regulation of rat uterine weight (17). Therefore, we hypothesized that changes in sex hormones during the menstrual cycle could lead to changes in pain perception and the interaction between sex hormones may be related to pain perception.

Because the menstrual cycle is characterized by dramatic changes in sex hormone levels, a large amount of experimental research on hormone–pain relationships has used the menstrual phase as a proxy for intraindividual variability in sex hormones (18, 19). There are many studies focused on experimental pain perception across the menstrual cycle in normal healthy females, but they report mixed results (20–25). The reasons for these inconsistencies may be as follows. First, the types of pain tested varied, for example, needle pain is likely A-delta fiber (AMH-I) mediated, pressure pain is mediated by C-nociceptors innervating deep tissue (26), and the sensation of cold pain is proposed to be mediated by transient receptor potential A1 (TRPA1) (27). Second, the phase of the menstrual cycle was not accurately defined. Furthermore, only the linear relationships between sex hormones and pain perception were analyzed, with no consideration of the complex interactions between them.

In addition, this research is different from those of previous studies in several ways. First, the menstrual cycle subphase is indeed more accurate with the addition of abdominal gynecological ultrasound monitoring to the protocol. Second, this study adhered to strict inclusion and exclusion criteria and added several exclusion criteria. Furthermore, six sex hormones were included for regression analysis, and two models (sex hormones/pain perception_ac_, sex hormones/pain perception_rc_) for sex hormone or pain perception changes in two subphases were established. The relationships between changes in sex hormones and pain perception and the interactions between sex hormones are presented here for the first time.

To verify the hypothesis, we recruited healthy young women. Menstrual days, gynecologic ultrasound, and gonadal hormones were used to determine the menstrual subphase. The main aim of this research was to investigate the differences in pain perception between the early to midfollicular phase (emF) and the midluteal phase (mL). The relationship between the changes in sex hormones and the change in pain perception and the effects of sex hormone interactions on pain perception was further explored.

## METHODS

### Subjects

Subjects were recruited from a group of medical students at Zhengzhou University People’s Hospital after approval of the Ethics Committee of the Zhengzhou University People’s Hospital (Ethics:2021, Ethical review 166 and the registration number is ChiCTR2100054783). Each subject voluntarily signed the informed consent form. This clinical trial registration number is ChiCTR2100054783.

From January 1, 2022, to May 31, 2022, based on the initial inclusion criteria, 80 volunteers were enrolled in the trial; 14 of those were later excluded (2 skin abnormality, 2 history of surgery,1 analgesic drug, 3 Pittsburgh sleep quality index >5, 2 hospital anxiety and depression scale >7, 2 pain catastrophizing scale ≥ 17, and 2 no ovulation). Sixty-six women are included in the final analyses. The inclusion criteria were as follows: age 22–28 yr with a regular menstrual cycle of 26–30 days, unmarried, no history of pregnancy, healthy nonsmokers, free of clinical pain, no substance abuse, no gynecological problems, and not having participated in other clinical trials in the past 3 mo.

Participants were excluded if they met any of the following criteria: skin abnormalities in the areas to be tested; pain catastrophizing scale (PCS) ≥ 17, hospital anxiety and depression scale (HADS) > 7, Pittsburgh sleep quality index (PSQI) > 5 history of surgery; trauma; analgesics or central drugs were used during the last month; abnormal pelvic ultrasound images; luteal-phase deficiency, when luteal phase <10 days and a midluteal progesterone (P) concentration <10 ng/mL; or anovulation.

### Determination of the Menstrual Cycle Phase

Each subject was tested twice with an interval of more than one menstrual cycle between both testing sessions. This interval between both test sessions was aimed at removing pain memories.

**Figure 1.**
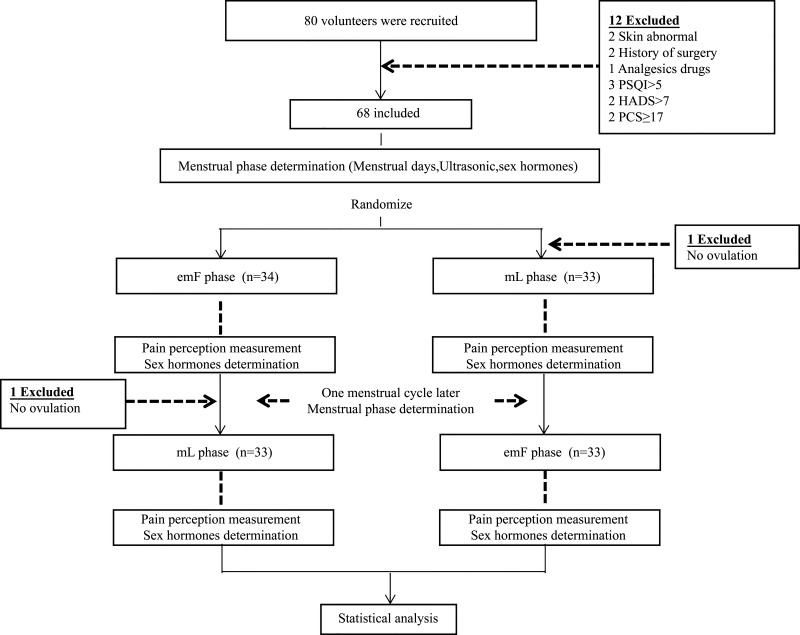
Study flow chart. emF, early to midfollicular phase; HADS, hospital anxiety and depression scale; mL, midluteal phase; PCS, pain catastrophizing scale; PSQI, Pittsburgh sleep quality index.

Serum gonadal hormone levels, ultrasonographic screening, and menstrual days were assessed to validate the cycling phase (Fig. 1). The first day of menses was considered *day 0*, subjects were tested once in the early to midfollicular subphase (emF) (defined as *days 4–9* from the onset of menses), and the midluteal subphase (mL) [sessions were scheduled for approximately 1 wk (±2–3 days) from the day of ovulation]. Pelvic ultrasound was used to monitor ovulation and ensure the emF and mL (see *Ultrasonographic procedures*). The time of the first testing period within the menstrual cycle was randomized between volunteers.

### Questionnaires

After determining the time of first participation and signing the informed consent form and before pain testing, the subjects completed a series of health and psychological questionnaires that included a collection of general information from the subjects, the pain catastrophizing scale (PCS), hospital anxiety and depression scale (HADS), and the Pittsburgh sleep quality index (PSQI).

### Measurements

All sessions were conducted by two experimenters (1 female and 1 male) who were blinded to the menstrual subphase. One experimenter interacted with participants, while the other operated the equipment and recorded the data. All tests begin at 2 PM and end at 4 PM. Before the test began, the subjects were acclimatized and verbal test instructions were given. The pain threshold test was composed mainly of a pain threshold perception test and a pain tolerance test. Pain threshold was defined as the first felt pain, and pain tolerance was defined as the point where the individual no longer felt able to tolerate the pain. The participants verbally indicated the feeling of pain in both assessments, and when the pain became intolerable, the data were recorded, and the experiment was ended. Pressure pain was recorded as final pressure (N), and cold and ischemic pain were recorded as persistent times (s). The pain of venipuncture was scored using numeric rating scales (NRS). On a scale of 1–10 points, 0 indicated that the participant was free of pain, and 10 indicated unbearable pain. Participants were asked to provide their scores after the needle had been withdrawn. The thresholds of pressure pain, cold pain, and ischemic pain were measured successively at an interval of 15 min, to reduce the impact of repeated tests on each other.

#### Blood sample collection.

The brachial vein of the elbow was selected for blood collection to detect the level of plasma sex hormones performed by the same experienced nurse at the end of each test. Blood was collected into the required number of vacutainer tubes using a standard 20-gauge butterfly needle.

As soon as the needle had been withdrawn and the puncture site swabbed, the subject-reported pain scores were obtained according to the NRS. In addition, the results of the NRS assessment were represented as needle pain (NP).

#### Pressure pain procedures.

Pressure pain was assessed using a handheld PainTest FPX 25 algometer (Wagner Instruments). The mechanical pressure was applied with a 1-cm^2^ probe. A deliberate application rate of 2 N/s was used to minimize reaction time artifacts. Subjects were asked to report when the pressure first became painful. Pressure pain threshold (PPTh) (N), which was defined as the threshold of perceived pain, was assessed three times at each anatomical site to obtain mean pressure ratings, and pressure pain tolerance (PPTo) (N), defined as the maximum pain tolerance, was assessed with the same methods, the interval between each repeat was 2 min. We also calculated the range between PPTo and PPTh (PPTo − PPTh) (N), referred to as the range of pain tolerance (RPT). The sites tested were as follows: center of the right trapezius (posterior to the clavicle) (*position 1*); right masseter (approximately midway between the tragus of the ear and the corner of the mouth) (*position 2*); the right ulna (dorsal forearm, ∼8 cm distal to the elbow) (*position 3*); and the pulp of the middle finger of the right hand (*position 4*).

#### Cold pain procedures.

As described in the previous literature (28, 29), subjects placed their nondominant hand in 0.1°C–1°C ice water, up to the wrist, without touching the bottom of the container. Two pain thresholds were recorded: the cold pain threshold (CPTh) (s) (from the time the participants put their hands into the water to the time they felt the pain) and cold pain tolerance (CPTo) (s) (from CPTh to CPTo keep recording until the time at which the pain became unbearable). When the hand was in the container, the water was constantly stirred to avoid local water temperature changes caused by body temperature affecting the measurement effect. The maximum measurement time was 3 min.

#### Ischemic pain procedures.

According to a previous study (20), the right arm of the subjects was exsanguinated by elevating it above the heart level for 30 s, after which the arm was occluded with a standard blood pressure cuff positioned proximal to the elbow and inflated to 200 mmHg using a mercury sphygmomanometer (Yuyue, China, Shanghai instrument registration 20162200750). Subjects then performed 20 handgrip exercises of 2-s duration at 4-s intervals at 50% of their maximum grip strength. Subjects were asked to report when they first felt pain, defined as ischemic pain threshold (IPTh) (s), and then to continue until the pain became intolerable, defined as ischemic pain tolerance (IPTo) (s). Then the time points were recorded.

#### Ultrasonographic procedures.

An ultrasound physician with 11 yr of working experience performed the ultrasound examination. Among the subjects participating in the project, an ultrasound examination was performed daily to determine the ovulation date and the menstrual cycle phase. The cycle phase was determined by ultrasound-based monitoring of normal follicular growth and ovulation (30, 31). The phase-specific ultrasound imaging features were as follows: the follicular phase was indicated by the appearance of dominant follicles and a thin homogenous echo in the endometrium; ovulation was indicated by the first appearance of dominant follicles measuring 16–28 mm in diameter, with their subsequent disappearance with or without detectable fluid in the pouch of Douglas; and after ovulation, the luteal phase was indicated by a thick endometrial (31) (Supplemental Fig. S1; see https://www.zenodo.org/record/8078760).

#### Hormone assays.

Blood samples were measured using a microparticle chemiluminescence immune analyzer (Beckman UniCel Dxi 800) at the end of each experiment. The plasma estradiol (E2), progesterone (P), follicle-stimulating hormone (FSH), luteinizing hormone (LH), prolactin (PRL), and testosterone (T) levels were measured.

### Sample Size Calculation and Statistical Analysis

The sample size was determined according to the rule of thumb that EPV (events per variable) equals 10; this rule of thumb refers to that at least 10 events per variable analyzed were desirable to maintain the validity of the model (32). There were six variables in total, and the minimum sample size was calculated by multiplying variables plus 10 (6 × 10 = 60) in a multifactor linear regression model.

The measurement data of the general characteristics and sex hormones, PPThs, PPTos, and RPTs across two menstrual cycle subphases were compared by paired *t* test or rank-sum test. The χ^2^ test was used for counting data. Statistical significance was defined as *P* < 0.05.

Two different data models were used in the correlation analysis and multivariate linear regression analyses of pain perception and sex hormones. The first model assessed the difference in pain perception or sex hormones between menstrual cycle subphases, defined as absolute changes in pain perception or sex hormones (pain perception/sex hormone_ac_), and the formula is as follows: pain perception/sex hormone_ac_ = pain perception/sex hormone_mL-emF_. The other model assessed the magnitude of changes in pain perception or sex hormones between menstrual subphases, defined as relative changes in pain perception or sex hormones (pain perception/sex hormone_rc_), and the formula is as follows: pain perception/sex hormone_rc_ = pain perception/sex hormone_(mL-emF)/emF_.

Multivariate linear regression analysis was performed on the pain perception_ac_ factors showing significant differences between the two menstrual cycles subphase and sex hormone_ac_. To investigate the relationship between the sex hormone_rc_ for six sex hormones and pain perception_rc_, we conducted correlation analysis and multivariate logistic linear between pain perception_rc_ and sex hormone_rc_. In the multivariate linear regression, we conducted interaction analysis when two or more factors (sex hormone_rc_) were found to be related to the dependent variable (pain perception_rc_). After the cutoff point was selected, the variable factors and interaction terms of the two or more factors were included in the model to analyze the interaction results. Restricted cubic spline (RCS) was used to determine the cutoff point in the interaction analysis. Statistical analysis was conducted using R language 3.6.3 and SPSS 26.0. Significance was set at *P* < 0.05.

## RESULTS

### General Characteristics

The general characteristics of participants are shown in Table 1. There was no statistical difference in the questionnaire scores of the subjects during the emF and mL (Table 2, *P* > 0.05).

**Table 1.** Demographic and clinical characteristics of participants

Characteristics	*n*	Mean (OR %)	SD	Range (Min, Max)
Age, yr	66	25.91	1.83	22, 28
BMI	66	20.87	1.37	18.50, 23.10
Level of education, %				
Bachelor	50	75.76		
Graduate students	16	24.24		
Religious beliefs, %				
Yes	2	3.03		
No	64	96.97		
Cycle length	66	28.91	1.28	26, 30
Menarche age, yr	66	13.09	0.84	11, 15

BMI, body mass index.

**Table 2.** Comparison of questionnaire differences between two menstrual subphases

Characteristics	emF	mL	Statistic	*P*
PSQI	4 (2.5, 5)	4 (3, 5)	*z* = −0.879	0.380
HAD-A	3.758 (2.151)	3.697 (2.271)	*t* = 0.121	0.857
HAD-D	3.73 (2.831)	3.82 (2.430)	*t* = −0.234	0.816
PCS	6 (1.5, 10)	5 (1, 12)	*z* = −1.075	0.283

Data conforming to normal distribution were represented by means (SD), whereas nonconforming data were represented by median (interquartile range, IQR); Chi-square test was used for counting data, paired *t* test was used for measurement data conforming to normal distribution, and rank-sum test was used for non-normal distribution (*n* = 66). emF, early to midfollicular phase; HAD-A, hospital anxiety and depression scale-anxiety score; HAD-D, hospital anxiety and depression scale-depression score; mL, midluteal phase; PCS, pain catastrophizing scale score; PSQI, Pittsburgh sleep quality index score.

### Hormone Levels in the emF and mL Phases

**Figure 2.**
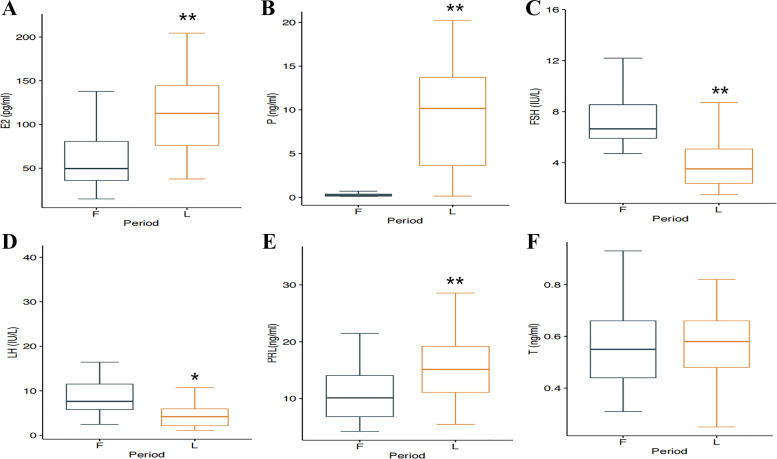
Plasma levels of sex hormones in the early to midfollicular (emF) and midluteal (mL) phases. *A*: change of E2 between emF and mL analyzed by paired *t* test measures. *t* = −5.973, **P < 0.01 vs. the emF. *B*: change of P between emF and mL analyzed by paired *t* test measures. *t* = −9.169, **P < 0.01 vs. the emF. *C*: change of FSH between emF and mL analyzed by paired *t* test measures. *t* = 5.426, **P < 0.01 vs. the emF. *D*: change of LH between emF and mL analyzed by Wilcoxon test of paired samples. *z* = 2.46, *P < 0.05 vs. the emF. *E*: change of PRL between emF and mL analyzed by paired *t* test measures. *t* = −5.034, **P < 0.01 vs. the emF. *F*: change of T between emF and mL analyzed by paired *t* test measures. *t* = −0.315, *P* = 0.755. *n* = 66. E2, estradiol; F, early to midfollicular phase; FSH, follicle-stimulating hormone; L, midluteal phase; LH, luteinizing hormone; P, progesterone; PRL, prolactin; T, testosterone.

The levels of all sex hormones, with the exception of testosterone, changed significantly between the emF and mL (Fig. 2). Compared with their levels in the emF, plasma estradiol (E2), progesterone (P), and prolactin (PRL) were increased to ∼1.86-fold (*P* < 0.01), 31.1-fold (*P* < 0.01), and 1.45-fold (*P* < 0.01), respectively, in the mL (Fig. 2, *A*, *B*, and *E*). In contrast, follicle-stimulating hormone (FSH) and luteinizing hormone (LH) were decreased to ∼0.57-fold (*P* < 0.01) and 0.80-fold (*P* < 0.05), respectively, in the mL (Fig. 2, *C* and *D*). Testosterone levels were similar in both subphases (*P* = 0.755) (Fig. 2*F*).

### Pain Thresholds and Tolerance in the Follicular and Luteal Phases

**Figure 3.**
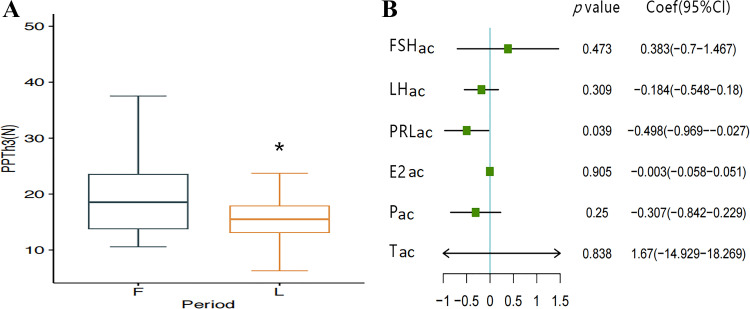
Differences in pain perception between the early to midfollicular (emF) and midluteal (mL) phase, and the effect of sex hormones. *A*: change of pressure pain in the right ulna pain threshold (PPTh3) between emF and mL phase analyzed by paired *t* test measures. *t* =2.231, *P < 0.05 vs. the emF. *B*: forest plot of the relationship between sex hormones_ac_ and PPTh3_ac_. Prolactin_ac_ between mL and emF phase related to the PPTh3_ac_ between two menstrual subphases analyzed by multivariate linear regression (β = −0.498, *P* = 0.039) (*n* = 66). E2_ac_, absolute change of estradiol; F, early to midfollicular phase; FSH_ac_, absolute change of follicle-stimulating hormone; L, midluteal phase; LH_ac_, absolute change of luteinizing hormone; P_ac_, absolute change of progesterone; PRL_ac_, absolute change of prolactin; sex hormones_ac_, absolute change of sex hormones; T_ac_, absolute change of testosterone.

PPThs, PPTos, CPTh, CPTo, IPTh, IPTo, and pinprick-evoked pain (PEP) were compared between menstrual cycle subphases. There was no significant difference in CPTh (*P* = 0.559), CPTo (*P* = 0.636), IPTh (*P* = 0.544), IPTo (*P* = 0.558), or PEP (*P* = 0.222) between the emF and mL (Table 3). For pressure pain, only the pressure pain threshold of the right ulna (PPTh3) was significantly different between the two cycles (*P* = 0.033) (Fig. 3*A*). There were no statistically significant differences between the threshold and tolerance values for other tenderness (*P* > 0.05) (Table 3).

**Table 3.** Pain Sensitivity in the emF and mL

Pain Procedures	Acronym	emF	mL	Change of Sensitivity mL vs. emF	Statistic	*P* Value
Pressure Pain, N						
Threshold of right trapezius	PPTh1	11.827 (6.303)	10.925 (6.507)	0.924	*t* = 1.206	0.222
Tolerance of right trapezius	PPTo1	20.101 (10.024)	20.217 (9.908)	1.006	*t* = −0.089	0.929
Threshold of right masseter	PPTh2	4.75 (3.95, 6.1)	4.275 (3.3, 5.7)	0.9	*z* = 0.822	0.242
Tolerance of right masseter	PPTo2	7.275 (6.075, 9)	7 (5.4, 8.35)	0.962	*z* = 0.259	0.796
Threshold of right ulna	PPTh3	19.341 (6.842)	16.706 (7.58)*	0.864	*t* = 2.231	0.033
Tolerance of right ulna	PPTo3	27.867 (10.052)	25.327 (9.188)	0.909	*t* = 1.421	0.165
Threshold of the pulp of the middle finger of the right hand	PPTh4	14.467 (11.767, 19.45)	13.05 (10, 17.467)	0.902	*z* = 1.796	0.073
Tolerance of the pulp of the middle finger of the right hand	PPTo4	25.979 (8.351)	24.307 (9.004)	0.936	*t* = 1.065	0.295
Cold pain, s						
Threshold	CPTh	6.765 (6.342)	7.238 (4.905)	1.07	*t* = −0.59	0.559
Tolerance	CPTo	15.087 (9.084)	15.639 (7.405)	1.037	*t* = −0.478	0.636
Ischemic pain, s						
Threshold	IPTh	19.01 (1.58,38)	13.73 (6.25,34.15)	0.722	*z* = 0.608	0.544
Tolerance	IPTo	79.001 (45.473)	83.962 (49.919)	1.063	*t* = −0.593	0.558
Needle pain (NRS score)	NP	3 (2,4)	4 (2,5)	1.333	*z* = 3.413	0.222

Data conforming to normal distribution were represented by means (SD), measured with paired *t* test, whereas nonconforming data were represented by median (IQR), measured with rank sum test; *P < 0.05 vs. the emF. emF, early to midfollicular phase; mL, midluteal phase; NRS, numeric rating scale.

### The Range of Pain Tolerance in the emF and mL Phases

We defined the range of pain tolerance (RPT) as the range between the pain tolerance and the pain threshold (RPT = pain tolerance − pain threshold) and found a significant correlation between pain threshold and pain tolerance in the emF and mL (Supplemental Table S1; see https://www.zenodo.org/record/8078760), and there was no significant difference in the RPT between the emF and mL (all *P* > 0.05) (Supplemental Table S2; see https://www.zenodo.org/record/8078760).

### Relationship between the Differences in PPTh3 and the Differences in Sex Hormone Levels between the Two Cycles

PPTh3 was significantly different between the mL and emF phases (*P* = 0.033) (Fig. 3*A*). We continued to analyze the relationship between PPTh3_ac_ and sex hormone_ac_ by multivariate linear regression. Figure 3*B* presents a forest plot of the relationships between sex hormones_ac_ and PPTh3_ac_. Prolactin_ac_ (PRL_ac_) was related to PPTh3_ac_ (OR = −0.498, *P* = 0.039), signifying that higher levels of PRL_ac_ were related to lower PPTh3_ac_.

### Relationship between Pain Perception_rc_ and Sex Hormone_rc_

We hypothesized that the relative change of the sex hormones (sex hormones_rc_) may also be an important factor affecting the relative change of the pain perception, so we further explored the relationship between pain perception_rc_ and sex hormone_rc_ by correlation analysis (Supplemental Table S3; see https://www.zenodo.org/record/8078760). It was found that estradiol_rc_ (E2_rc_) was correlated with the relative change of the range of pain tolerance of pressure pain of right masseter (RPT2_rc_), the relative change of the pressure pain threshold of the pulp of the middle finger of the right hand (PPTH4_rc_), and the relative change of pressure pain tolerance of the pulp of the middle finger on the right hand (PPTo4_rc_). Specifically, higher E2_rc_ was associated with higher RPT2_rc_ (*r* = 0.185, *P* = 0.04, Supplemental Table S3), higher PPTh4_rc_ (*r* = 0.06, *P* = 0.031, Supplemental Table S3), and higher PPTo4_rc_ (*r* = 0.067, *P* = 0.03, Supplemental Table S3). In addition, progesterone_rc_ (P_rc_) was a significantly associated with the relative change of the range of pain tolerance for pressure pain of the right ulna (RPT3_rc_). Higher P_rc_ was also associated with higher RPT3_rc_ (*r* = 0.013, *P* = 0.016, Supplemental Table S3).

**Figure 4.**
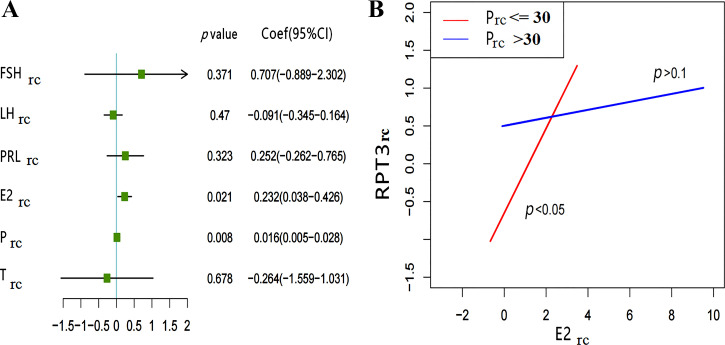
Relationship between the RPT3_rc_ and sex hormones_rc_ levels. *A*: forest plot of the relationship between sex hormones_rc_ and RPT3_rc_. The P_rc_ (β = 0.016, *P* = 0.008) and E2_rc_ (β = 0.232, *P* = 0.021) related to RPT3_rc_, analyzed by multivariate linear regression. *B*: results of interaction between effect of P_rc_ and E2_rc_ on RPT3_rc_. P_rc_= 30 is the cutoff point selected in interaction analysis, the red line represents P_rc_ ≤ 30; E2_rc_ related to change in RPT3_rc_ (*P* < 0.05), the blue line represents P_rc_ > 30, the change of RPT3_rc_ is independent of E2_rc_ (*P* > 0.1) (*n* = 66). E2_rc_, relative change of estradiol; FSH_rc_, relative change of follicle-stimulating hormone; LH_rc_, relative change of luteinizing hormone; P_rc_, relative change of progesterone; PRL_rc_, relative change of prolactin; RPT3, pain tolerance range for right ulna tenderness; RPT3_rc_, relative change of RPT3; sex hormones_rc_, relative change of sex hormones; T_rc_, relative change of testosterone.

**Figure 5.**
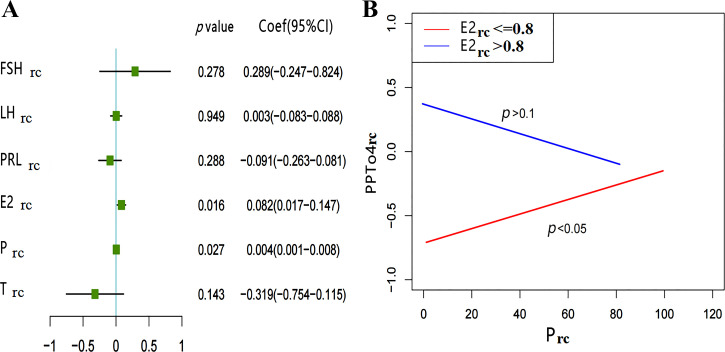
Relationship between the PPTo4_rc_ and sex hormones_rc_ levels. *A*: forest plot of the relationship between sex hormone_rc_ and PPTo4_rc_. E2_rc_ (β = 0.082, *P* = 0.016) and P_rc_ (β = 0.004, *P* = 0.027) related to PPTo4_rc_, analyzed by multivariate linear regression. *B*: results of interaction between effect of E2_rc_ and P_rc_ on PPTo4_rc_. E2_rc_ = 0.8 is the cutoff point selected in interaction analysis, the red line represents E2_rc_ ≤ 0.8; P_rc_ related to the change in PPTo4_rc_ (*P* < 0.05), the blue line represents E2_rc_ > 0.8, the change of PPTo4_rc_ is independent of P_rc_ (*P* > 0.1) (*n* = 66). E2_rc_, relative change of estradiol; FSH_rc_, relative change of follicle-stimulating hormone; LH_rc_, relative change of luteinizing hormone; P_rc_, relative change of progesterone; PPTo4, right middle finger pressure pain tolerance; PPTo4_rc_, relative change of PPTo4; PRL_rc_, relative change of prolactin; sex hormones_rc_, relative change of sex hormones; T_rc_, relative change of testosterone.

Furthermore, the relationship between sex hormone_rc_ and pain perception_rc_ (RPT2_rc_, RPT3_rc_, PPTH4_rc_, and PPTo4_rc_) was analyzed by multivariate linear regression with the inclusion of multiple sex hormones_rc_. We found E2_rc_ and P_rc_ to be related to RPT3_rc_ or PPTo4_rc_. In addition, higher E2_rc_ (β = 0.232, *P* = 0.021) and higher P_rc_ (β = 0.016, *P* = 0.008) were associated with higher RPT3_rc_ (Fig. 4*A*). Similarly, higher E2_rc_ (β = 0.082, *P* = 0.016) and higher P_rc_ (β = 0.04, *P* = 0.027) were associated with higher PPTo4_rc_ (Fig. 5*A*). There were no significant relationships between sex hormones_rc_ and RPT2_rc_ (all *P* > 0.05) or between sex hormones_rc_ and PPTh4_rc_ (all *P* > 0.05). (Supplemental Fig. S2; see https://www.zenodo.org/record/8078760).

To further explore the effect of sex hormone_rc_ interactions on pain perception_rc_, we changed P_rc_ into a categorical variable and explored the effect of E2_rc_ on RPT3_rc_ at different P_rc_, finding that E2_rc_ (*P* = 0.00561), P_rc_ (*P* = 0.00756), and their interaction terms (*P*-interaction = 0.02176) were significantly related to the outcome (RPT3_rc_). When P_rc_ was low (≤30), E2_rc_ was positively correlated with RPT3_rc_ (*P* < 0.05), but when P_rc_ was high (>30), there was no significant correlation between E2_rc_ and RPT3_rc_ (*P* > 0.1) (Fig. 4*B*).

With the same method, we changed E2_rc_ into a categorical variable and explored the effect of P_rc_ on PPTo4_rc_ at different E2_rc_, finding that E2_rc_ (*P* = 0.000582), P_rc_ (*P* = 0.005483), and their interaction terms (*P*-interaction = 0.044336) were significantly related to the outcome (PPTo4_rc_). We found that when E2_rc_ was low (≤ 0.8), P_rc_ was positively correlated with PPTo4_rc_ (*P* < 0.05) but that when E2_rc_ was high (>0.8), there was no significant correlation between P_rc_ and PPTo4_rc_ (*P* > 0.1) (Fig. 5*B*).

## DISCUSSION

The current study examined the relationships between sex hormones and pain perception during the menstrual cycle in healthy young women. The results revealed that the absolute change of ulna threshold in pressure pain perception (PPTh3_ac_) was significantly related to changes in the absolute change of PRL levels across the menstrual cycle subphase. The conclusion that PPTh3 has a lower pain threshold in the mL, that is, higher pain sensitivity, is consistent with the conclusions of some studies (26, 33). In addition, we found that higher PRL_ac_ was related to lower PPTh3_ac_ across the menstrual subphases. This is consistent with the conclusion that PRL promotes nociceptor sensitization and pain in women (34).

To our knowledge, our study is the first to find that the relative change of pain perception (pain perception_rc_) across the menstrual cycle subphase is related to the relative change of sex hormone levels (sex hormones_rc_) and that the different sex hormones interact with each other. After we established the pain perception_rc_ and sex hormone_rc_ model, the more interesting finding was that E2_rc_ and P_rc_ were correlated with RPT3_rc_ and PPTo4_rc_, and E2_rc_ interacts with P_rc_. Regarding at different P_rc_, the correlation between E2_rc_ and RPT3_rc_ was positive (*P* < 0.05) or nonexistent (*P* > 0.1). Similarly, for PPTo4_rc_, P_rc_ was either positively (*P* < 0.05) or not correlated (*P* > 0.1) with PPTo4_rc_ at different E2_rc_. This supports the idea that the net effect of sex hormones on pain is complex (19) and that the correlation between the relative change of E2/P and PPTo4/RPT3 may depend on the degree of hormone interaction and fluctuation. We speculate that the interaction between the two sex hormones may be related to competition between receptors (35). As an exploratory finding, its clinical significance is unclear and still needs further replication to prove its scientific validity.

The experimental pain model was used to investigate the difference in pain perception between the emF and the mL and the role of sex hormones. These models have been shown to be of great value in clinical studies for quantifying the sensitivity of the pain sensation system in patients with pain and for predicting clinical outcomes (36–38). Since responses to different types of pain are often uncorrelated, and menstrual phase, segmental site, tissue depth, and sex all have unique interacting effects on pain thresholds, it is best to use multiple pain types in studies of pain thresholds (39–41). Therefore, in this study, we selected pressure pain in different site, needle pain, cold pain, and ischemic pain. We chose these because the mechanism of needle pain is similar to that of incisional pain and may be mediated by the A-delta fiber (AMH-I), and cold pain and muscle ischemic pain are thought to be the most clinically relevant types of pain (19, 26).

In studies of the relationship between menstrual cycle subphases and pain perception in healthy females, the definition of menstrual subphase is important (42). Many studies did not verify the menstrual phase by confirming that the participants had ovulated but only simply counted days starting with the first day of menses (43–47); this fails to account for the wide variability in menstrual cycle duration and does not confirm that ovulation has occurred (16). In this study, in addition to recording the days of menstruation and hormone measurement, gynecological ultrasound was used to examine ovulation, follicular size, and endometrial thickness to determine the menstrual cycle stage (30, 31), and listed the luteal-phase deficiency as exclusion criteria, when luteal phase < 10 days and a midluteal P concentration < 10 ng/mL (48). Thus, the menstrual cycle status determination was more accurate.

Studies have shown that sleep quality, anxiety, depression, and pain catastrophizing perception all affect pain perception (49–52). Abnormal HADS, PSQI, and PCS scores were used as exclusion criteria in this study to ensure the uniformity of included subjects. In this study, we found no cycle phase-dependent changes in the HADS, PSQI, or PCS scores, although previous studies have reported significant changes in emotional symptoms across the menstrual cycle (53, 54). The possible reason is that subjects with abnormal HADS, PSQI, and PCS scores (PCS ≥ 17, HADS > 7, or PSQI > 5) were excluded.

Pain threshold is divided into perceived pain threshold (pain threshold) and pain tolerance threshold (pain tolerance). The pain threshold is relatively constant and is less influenced by psychological and psychosocial factors, while tolerance may constitute a learned component of pain that is a more sensitive index of psychological, motivational, and cultural factors affecting the experience of pain (55). However, the relationship between the difference value of the two and the menstrual cycle was rarely observed. We believe that the different value of the two reflects the range of individual pain tolerance, which is defined as the RPT in this study. Also in this study, one interesting finding was that pain thresholds for all pain types were strongly correlated with pain tolerance. Although no difference in RPT was found between the emF and mL in this study, we found that the P_rc_ and E2_rc_ related to RPT3_rc_. The relationship between the RPT_rc_ and the sex hormone_src_ was analyzed for the first time.

### Perspectives and Significance

In this study, we found that some types of sex hormone fluctuations are associated with certain types of pain perception in healthy young women in an interactive way. Exploration of the interplay between sex hormones may hold the key to understanding the relationship between sex hormones and pain perception.

### Limitations

There are several limitations to this study. First, females with specific physical conditions were also excluded from the study, so it is unclear what the results are for puberty, menopause, or pregnant women. And this study involved only two points during the menstrual cycle. Further study of the late follicular subphase, the ovulatory subphase, and the late-luteal (or premenstrual) subphase is necessary.

Second, in the selection of pain types, only clinically relevant pain types were selected, but for ethical reasons, there were restrictions regarding some pain types, such as incisional pain, visceral pain, and heat pain, which were not included in the selection of pain types; therefore, whether our conclusion can be verified in other pain types needs further study.

Third, the effects of hydration on pain perception have also been reported, and hydration may also be linked to changes in the phases of the menstrual cycle (42, 56, 57). This study mainly observed the correlation between changes in sex hormones and changes in pain threshold but not including hydration in the analysis, which is also one of the limitations of this study.

Finally, we carried out two kinds of formula transforms models (absolute change and relative change), and the results did not have a consistent pattern. In general, most pain tests showed no difference between the two subphases of the menstrual cycle. Although this is consistent with our previous prediction, it also shows that the correlation between sex hormone changes and pain perception in women during different menstrual subphases is very complex. It is urgent to establish the physiological or theoretical rationale for it. In addition, although the results are statistically significant in this study, their biological significance needs more further research.

### Conclusions

Two different models of sex hormones and pain perception were used in this study and showed inconsistent results. Most pain tests showed no difference between the two subphases of the menstrual cycle. In the first model of absolute change, we found that changes in the right ulna pressure pain threshold between menstrual subphase could be related to PRL_ac_. PRL may be an important target for future study of pain perception in women between menstrual cycles. In the second model of relative change, we found that the relative change of the PPTo4 and RPT3 are related to the E2_rc_ and P_rc_, respectively, between menstrual subphase in an interactive way, demonstrating that the net effect of sex hormones on pain perception is complex.

## DATA AVAILABILITY

The raw data supporting the conclusions of this article will be made available by the authors, without undue reservation. The original contributions presented in the study are included in the article/Supplementary Material, further inquiries can be directed to the corresponding author.

## SUPPLEMENTAL DATA

Supplemental Figs. 1 and 2 and Supplemental Tables S1–S3: https://www.zenodo.org/record/8078760.

## GRANTS

This study was supported by the grant of the Henan Province Middle-aged and Young Health Science and Technology Innovation Outstanding Youth Talent Training Project (YXKC2021025 to W. Zhang) and the National Natural Science Foundation of China (81901110 to M. Sun).

## DISCLOSURES

No conflicts of interest, financial or otherwise, are declared by the authors.

## AUTHOR CONTRIBUTIONS

J.Z. and W.Z. conceived and designed research; L.Z., Y.Z., X.L., and J.C. performed experiments; Y.Z. and X.L. analyzed data; M.S. interpreted results of experiments; W.Z. edited and revised manuscript; W.Z. approved final version of manuscript.
